# Analysis of diastasis recti abdominis phenotypes and related delivery factors at 42 days postpartum

**DOI:** 10.1080/07853890.2025.2523556

**Published:** 2025-06-26

**Authors:** Jingjing Guo, Lingyan Liu, Min Hua, Dong Han, Xuezhen Tang, Jiying Wen, Yuheng Zhou

**Affiliations:** a Obstetric Department, Guangdong Women and Children Hospital, Guangzhou, China; b Department of Quality Control, The Third Affiliated Hospital of Southern Medical University, Guangzhou, China; c Ultrasound Department, Guangdong Women and Children Hospital, Guangzhou, China

**Keywords:** Pregnancy, childbirth, postpartum, diastasis recti abdominis, intra-abdominal pressure

## Abstract

**Objective:**

Diastasis recti abdominis (DRA) is highly prevalent in the postpartum period and affects women’s health. However, the relationship between DRA and prenatal and intrapartum factors is unclear. This study aimed to investigate DRA phenotypes and assess the influence of prenatal and intrapartum factors at 42 days postpartum.

**Materials and methods:**

This retrospective study included 1438 singleton primiparous women who delivered between January 2023 and January 2024 at a single institution. The median age was 29 years, and the median gestational age at delivery was 274 days. The exclusion criteria included factors that might affect intra-abdominal pressure during the pre-pregnancy and pregnancy periods. DRA was defined as an inter-rectus distance of ≥20 mm at any of the three measurement sites: 3 cm above/below the umbilicus and at the umbilicus, measured by ultrasound. The Kruskal-Wallis and chi-square tests were used for variance analysis and comparison of variables, respectively. Multivariate logistic regression analysis was performed to explore the relationships between DRA phenotypes and these factors.

**Results:**

The incidence of DRA was 89.4%, 47.8% of which presented as combined upper umbilical and periumbilical separation, 33.7% as isolated periumbilical separation, 6.7% as complete separation, and 10.6% without separation. The combined separation of the upper umbilical and periumbilical areas was associated with increased BMI, neonatal weight, vaginal delivery, episiotomy, and perineal laceration. Complete separation was associated with BMI, neonatal weight, and vaginal delivery. Isolated periumbilical separation was associated with neonatal weight, vaginal delivery, episiotomy, perineal laceration, and cesarean during the first stage of labor. Vaginal delivery was negatively correlated with DRA.

**Conclusion:**

DRA is highly prevalent at 42 days postpartum, with different phenotypes having distinct prenatal and intrapartum risk factors. Careful weight management during pregnancy, promotion of vaginal delivery, and rational use of perineal episiotomies may reduce the incidence of DRA in primiparous women.

## KEY MESSAGES

Vaginal delivery is a protective factor against postpartum diastasis recti abdominis (DRA). Factors such as increased BMI, neonatal weight, perineal episiotomy/laceration, and labor trial are associated with different DRA subtypes.

Perineal episiotomy/laceration may protect against lower umbilical separation, while labor trial in the first stage of labor may reduce upper umbilical separation.

Strategic weight management during pregnancy, encouragement of vaginal trial of labor, and judicious use of episiotomy are key strategies to reduce the incidence of DRA in postpartum women.

## Introduction

Diastasis recti abdominis (DRA) is characterized by a widening of the inter-rectus distance due to excessive stretching and relaxation of the linea alba, which connects the two muscles of the recti abdominis [1]. This condition not only affects body posture [2] but also leads to core dysfunction [3] which can cause low back pain, urinary incontinence [4], and reduced quality of life [5–7], resulting in long-term physiological and psychological effects [8].

DRA is closely related to pregnancy, with postpartum incidence rates ranging from 42% to 83% [9–11]. The umbilical region is the area most affected in women with DRA [12], and it is recommended that the inter-rectus distance be examined at the upper, periumbilical, and lower umbilical sites postpartum. Recent population surveys have identified five DRA phenotypes: isolated upper separation, isolated lower separation, periumbilical separation, upper gap-dominant complete separation, and lower gap-dominant complete separation [13]. The varying incidence rates of these phenotypes suggest that the mechanisms underlying DRA at different sites may differ from one another.

Elevated intra-abdominal pressure (IAP) is a cause of postpartum DRA [12,14]. The abdominal muscles, fascia, and peritoneum form the abdominal cavity, which maintains IAP [15]. Abdominal muscle contraction, respiratory movements, and abdominal contents can increase IAP [16,17]. Significant abdominal enlargement during pregnancy is closely related to DRA [18,19], however, the impact of cesarean and vaginal deliveries on the abdominal muscles is inconsistent [20]. From the perspective of IAP, for women undergoing direct cesarean sections, the pressure on the recti abdominis muscles during pregnancy mainly comes from the enlarged uterus and other abdominal organs, with gestational age, fetal size, amniotic fluid volume, and maternal weight being potential factors. For women attempting vaginal delivery, the impact on the recti abdominis muscles should be divided into prenatal and intrapartal stages of labor, respectively. The prenatal impact is consistent with that of women undergoing direct cesarean sections. However, during the intrapartum stage, uterine contractions are the primary force for fetal descent during the first stage of labor. In the second stage, the abdominal muscles actively contract to assist with fetal expulsion. Therefore, the factors influencing IAP differ between the delivery modes. The processes experienced by women who have spontaneous vaginal delivery, assisted delivery, or cesarean section after a trial of labor are not the same.

Currently, there is a lack of research on the relationship between prenatal and intrapartum factors and DRA. However, postpartum abdominal ultrasound screening and DRA-related therapy are conducted in many institutions, requiring significant human, material, and financial resources to undertake these procedures. Exploring the phenotypes of postpartum DRA and related prenatal and intrapartum factors can guide prenatal and intrapartum prevention strategies and optimize postpartum rehabilitation efforts, thereby enhancing the efficient use of resources.

Most physiological changes that occur during pregnancy take approximately six weeks to revert to their pre-pregnancy state. However, studies [21,22] have reported that the prevalence of DRA is approximately 33.1% in mid-pregnancy, reaches 100% in late pregnancy, decreases to approximately 60% at six weeks postpartum, and gradually declines to 32.6% by 12 months postpartum. Therefore, we selected 42 days postpartum as the assessment time point for this study. On the one hand, the high prevalence of DRA before childbirth makes it unsuitable for evaluating intrapartum factors, and after 42 days postpartum, DRA begins to recover gradually, which could interfere with the assessment of related factors. On the other hand, 42 days postpartum is a standard time point for postpartum follow-up, ensuring an adequate sample size for the study. Additionally, abdominal muscle separation screening and rehabilitation recommendations typically begin during this period.

This study aimed to understand the relationship between the types of DRA and prenatal factors (BMI gain and newborn weight) and perinatal factors (vaginal delivery, perineal episiotomy, perineal laceration, and emergency cesarean section during the first or second stage of labor) at 42 days postpartum. During the statistical analysis, we adjusted for the following factors: prenatal correction factors, including maternal age, gravidity, gestational age, and pre-pregnancy BMI (p-BMI), and perinatal correction factors, including assisted birth, precipitous labor, and labor analgesia.

## Materials and methods

This study adhered to the principles of the Declaration of Helsinki and was approved by the Ethics Committee of Guangdong Women and Children Hospital (Ethics Number: 202401202), with a waiver of informed consent.

### Data collection

This study was conducted at Guangdong Women and Children Hospital in Guangzhou City, China. A total of 2923 postpartum abdominal ultrasound reports were collected between January 2023 and January 2024. The inclusion criterion was singleton primiparity. The exclusion criteria were as follows: (1) multiparity; (2) multiple pregnancies; (3) history of missed or induced abortion between 20 and 28 weeks of gestation; (4) history of abdominal wall disease and/or hernia; (5) history of abdominal surgery; (6) chronic cough; (7) paraplegia; (8) abnormal amniotic fluid volume during the current pregnancy; (9) vertical abdominal incision in the current cesarean section; and (10) delivery outside the hospital. After further review of the medical records system, 223 cases of delivery outside our hospital, 21 cases of twin pregnancies, 1 case of triplet pregnancies, and 1240 cases of multiparity were excluded. The final number of cases included in the study was 1438.

**Figure 1.**
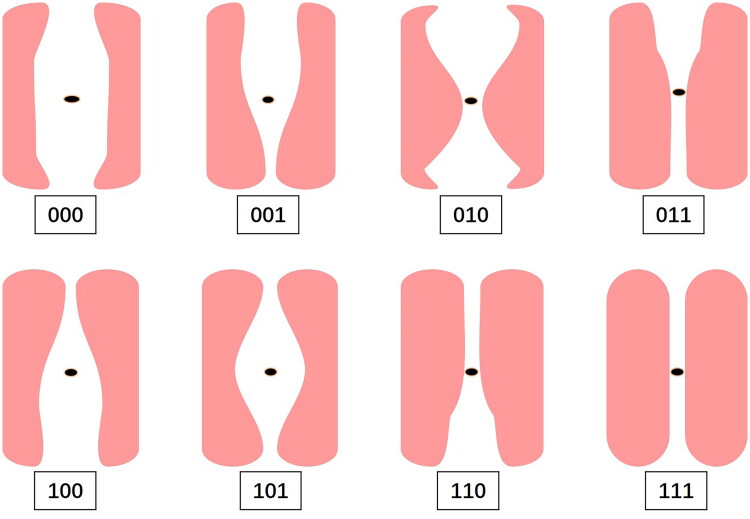
Possible phenotypes of postpartum diastasis recti abdominis in binary combinations. 000: complete separation type; 001: combined upper umbilical and periumbilical separation type; 010: combined upper umbilical and lower umbilical separation type; 011: isolated upper umbilical separation type; 100: combined periumbilical and lower umbilical separation type; 101: isolated periumbilical separation type; 110: isolated lower umbilical separation type; 111: no separation type.

Abdominal ultrasound measurements of the recti abdominis muscles were performed at three sites: 3 cm above the umbilicus, at the level of the umbilicus, and 3 cm below the umbilicus. DRA was diagnosed if the inter-rectus distance was ≥20 mm at any of these sites. The DRA phenotypes were expressed in binary form to clearly and intuitively display the separation patterns. Using ‘0’ to indicate separation and ‘1’ to indicate no separation, eight possible combinations were identified: complete separation type (000), combined upper umbilical and periumbilical separation type (001), combined upper umbilical and lower umbilical separation type (010), isolated upper umbilical separation type (011), combined periumbilical and lower umbilical separation type (100), isolated periumbilical separation type (101), isolated lower umbilical separation type (110), and no separation type (111) (Figure 1).

The following data were collected from the medical record system: age, last menstrual period, height, pre-pregnancy weight (p-weight), weight before delivery (d-weight), pre-pregnancy body mass index (p-BMI), BMI before delivery (d-BMI), gravidity, gestational weeks at delivery, mode of delivery, neonatal weight, assisted delivery, episiotomy, perineal laceration, time of cesarean section during labor trial (first or second stage), precipitous labor, and labor analgesia. Gestational age (days) was calculated based on the crown-rump length (CRL) measured by ultrasound at 11-13^+6 ^weeks of gestation. For example, if the gestational week was 40 weeks and 1 d, the gestational age (days) was calculated as 40 × 7 + 1 = 281 days. BMI gain during pregnancy was calculated by subtracting p-BMI from d-BMI. The modes of delivery were classified as cesarean section or vaginal delivery, with vaginal delivery encompassing spontaneous and assisted deliveries (forceps or vacuum extraction). If both episiotomies and lacerations were present, they were recorded as episiotomies only. The first stage of labor was defined as the period from the onset of labor to full cervical dilation. In this study, if oxytocin was used to induce contractions, regular contractions were considered the onset of labor, regardless of the cervical dilation. At our institution, if regular contractions did not commence within 1 h after artificial rupture of membranes, oxytocin was administered to induce labor. If induction failed after more than 12 h, the patient could choose to continue induction or switch to a cesarean section, according to her preference. The use of oxytocin at any stage of labor to regulate contractions was considered oxytocin-induced labor. The second stage of labor refers to the period from full cervical dilation to fetal expulsion. The cesarean sections were performed during trials of labor that spanned the first and second stages. When cervical dilation was ≥1 cm, labor analgesia was administered according to the patient’s preference.

### Postpartum ultrasound of recti abdominis

All ultrasound examinations in this study were performed using a GE Voluson E8 color Doppler ultrasound diagnostic instrument with a 5–12 MHz linear array probe. The examination procedure and standards followed the ‘Expert Consensus on Diagnosis and Treatment of Postpartum Diastasis Rectus Abdominis (2023)’ [23]. In the supine resting position (with relaxed abdominal muscles), the inter-rectus distance was measured at 3 cm above the umbilicus, at the umbilicus, and 3 cm below the umbilicus. DRA was diagnosed if the distance exceeded 2 cm at any of the three points of interest.

### Statistical analysis

Statistical analyses were performed using SPSS software (IBM, Version 26). The Shapiro-Wilk test was used for normality testing of continuous variables, and the Levene test was used for homogeneity of variance. As all variables were not normally distributed, continuous variables were described using the median and interquartile range (IQR). Categorical variables were expressed as numbers and percentages. The Kruskal-Wallis test was used for variance analysis, and categorical variables were compared using the chi-square test. Before the regression analysis, collinearity tests were performed on continuous and categorical variables, excluding independent variables with a variance inflation factor (VIF) of less than 5. The final independent variables were subjected to multivariate logistic regression analysis. Five multivariate logistic regression models were used, with DRA phenotypes as the dependent variable and no separation type (111) as the reference variable. Model 1 lists the odds ratios (OR) of each prenatal factor after including the prenatal correction factors. Model 2 presents the OR of each prenatal and intrapartum factor without correction for confounding factors. Model 3 included only prenatal correction factors for each prenatal and intrapartum factor. Model 4 included only intrapartum correction factors for each prenatal and intrapartum factor. Model 5 included all correction factors for each prenatal and intrapartum factor. Statistical significance was set at *p* < 0.05.

## Results

### Characteristics of the study population

All women included in this study were Chinese nationals. The median age was 29 (27, 30) years, the median gestational days at delivery was 274 (268, 280) days, the median weight gain during pregnancy was 13.4 (10.4, 16.5) kg, and the median neonatal birth weight was 3.1 (2.8, 3.4) kg. The vaginal delivery rate was 61.9%, the cesarean section rate during the first stage of labor was 14.4%, and the cesarean section rate during the second stage was 1.4%. No records showed that these women engaged in moderate-to-vigorous physical activity during pregnancy or the postpartum period, nor were they professional athletes during these periods (Table 1).

**Table 1.** Comparison of prenatal and perinatal data among subgroups with diastasis recti abdominis.

	Total (*n* = 1438)	000 (*n* = 96)	001 (*n* = 687)	101 (*n* = 484)	111 (*n* = 152)	*p*
Maternal age (years)	29 (27, 32)	30 (27, 32)	29 (27, 32)	29 (26, 31)	28 (26, 31)	0.029
Gravidity	1 (1, 2)	1 (1, 2)	1 (1, 2)	1 (1, 2)	1 (1, 2)	0.251
Gestational days (days)	274 (268, 280)	278 (271.5, 281)	275 (269, 280)	274 (267, 280)	272 (264, 277)	<0.001
Height (cm)	159 (156, 163)	159 (155, 163)	159 (156, 163)	159 (156, 163)	159 (155, 163)	0.549
p-Weight (kg)	52 (47, 57)	53 (48, 58.7)	52 (48, 57.5)	51 (46.6, 56)	50 (45.4, 55)	0.001
d-Weight (kg)	65.3 (60, 71.2)	68.4 (62.1, 74)	66.1 (60.9, 72.1)	64 (59, 69.7)	64 (58.3, 68.7)	<0.001
Weight gain (kg)	13.4 (10.4, 16.5)	14.4 (12.3, 18.6)	13.7 (10.7, 17)	13 (10, 16)	12 (9.5, 15.4)	<0.001
p-BMI	20.3 (18.8, 22.2)	21.3 (19, 23.3)	20.6 (19, 22.7)	20 (18.5, 21.7)	19.9 (18.7, 21.4)	<0.001
d-BMI	25.7 (23.9, 27.9)	27.3 (25, 29.3)	26.1 (24.3, 28.3)	25.3 (23.5, 27.3)	24.9 (23.3, 26.9)	<0.001
BMI gain	5.3 (4.1, 6.5)	5.8 (4.8, 7.3)	5.4 (4.3, 6.6)	5.1 (4.0, 6.4)	4.7 (3.7, 6)	<0.001
Neonatal weight (kg)	3.1 (2.8, 3.4)	3.2 (2.9, 3.5)	3.1 (2.8, 3.4)	3.1 (2.8, 3.3)	3 (2.7, 3.2)	<0.001
Vaginal delivery (%)	890 (61.9%)	43 (44.8%)	400 (58.2%)	318 (65.7%)	115 (75.7%)	<0.001
Cesarean (%)	548 (38.1%)	53 (55.2%)	287 (41.8%)	166 (34.3%)	37 (24.3%)	
Forceps (%)	121 (8.4%)	0	8 (1.2%)	5 (1%)	3 (2%)	0.261
Vacuum extraction (%)	16 (1.1%)	3 (3.1%)	61 (8.9%)	47 (9.7%)	7 (4.6%)	
Perineal episiotomy (%)	289 (20.1%)	11 (11.5%)	143 (20.8%)	99 (20.5%)	31 (20.4%)	<0.001
perineal laceration (%)	573 (39.8%)	31 (32.3%)	247 (35.95%)	215 (44.4%)	71 (46.7%)	
Precipitous labor (%)	26 (1.8%)	0	12 (1.8%)	10 (2.1%)	3 (1.97%)	0.41
ECS^1st^ (%)	207 (14.4%)	22 (41.5%)	105 (36.6%)	72 (43.4%)	7 (18.9%)	0.001
ECS^2nd^ (%)	20 (1.4%)	3 (3.1%)	12 (1.8%)	4 (0.8%)	1 (0.7%)	
Labor analgesia (%)	962 (66.9%)	58 (60.4%)	451 (65.7%)	334 (69.0%)	105 (69.1%)	0.312

000: complete separation type; 001: combined supraumbilical and periumbilical separation type; 101: isolated periumbilical separation type; 111: no separation type; ECS1st: cesarean section during the first stage of labor; ECS2nd: Cesarean section during the second stage of labor. *Notes*: Continuous variables are described using median and interquartile range, and categorical variables are expressed as counts and percentages. Continuous variables were analyzed using the Kruskal-Wallis test for variance analysis, and categorical variables were compared using the chi-square test.

### Postpartum diastasis recti abdominis phenotypes and proportions

**Figure 2.**
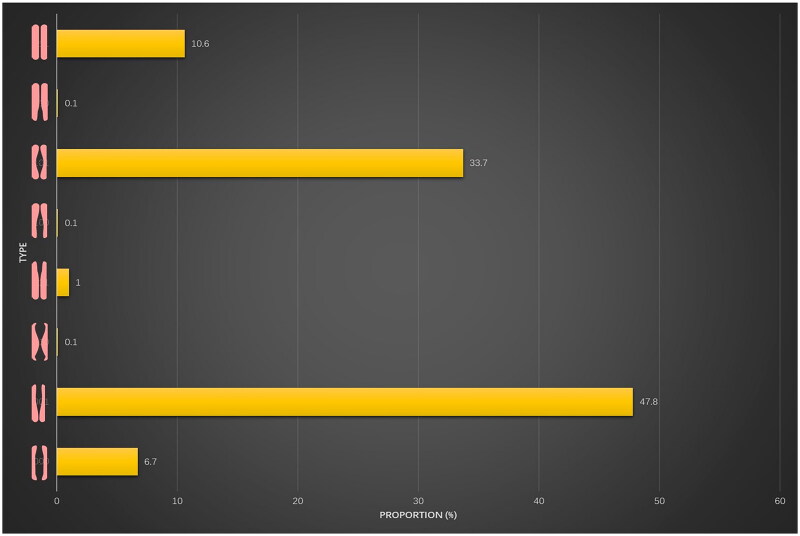
Postpartum diastasis recti abdominis phenotypes and proportions. 000: complete separation type; 001: combined upper umbilical and periumbilical separation type; 010: combined upper umbilical and lower umbilical separation type; 011: isolated upper umbilical separation type; 100: combined periumbilical and lower umbilical separation type; 101: isolated periumbilical separation type; 110: isolated lower umbilical separation type; 111: no separation type.

Among the 1438 postpartum women included in this study, 1286 cases (89.4%) were diagnosed with DRA. The phenotypes included complete separation type (000) in 96 cases (6.7%), combined upper umbilical and periumbilical separation type (001) in 687 cases (47.8%), combined upper umbilical and lower umbilical separation type (010) in one case (0.1%), isolated upper umbilical separation type (011) in 15 cases (1%), combined periumbilical and lower umbilical separation type (100) in two cases (0.1%), isolated periumbilical separation type (101) in 484 cases (33.7%), isolated lower umbilical separation type (110) in one case (0.1%), and no separation type (111) in 152 cases (10.6%). Owing to the small sample size, phenotypes 010, 011, 100, and 110 were excluded from the statistical analysis (Table 2; Figure 2).

**Table 2.** Postpartum recti abdominis phenotypes and proportions (*N* = 1438).

Type	Proportion (%)	*n*
000	6.7	96
001	47.8	687
010	0.1	1
011	1	15
100	0.1	2
101	33.7	484
110	0.1	1
111	10.6	152

000: complete separation type; 001: combined supraumbilical and periumbilical separation type; 010: combined supraumbilical and infraumbilical separation type; 011: isolated supraumbilical separation type; 100: combined periumbilical and infraumbilical separation type; 101: isolated periumbilical separation type; 110: isolated infraumbilical separation type; 111: no separation type.

### Comparison of prenatal and intrapartum data among different DRA phenotypes

Variance analysis revealed statistically significant differences in maternal age, gestational days, pre-pregnancy weight, weight before delivery, weight gain, pre-pregnancy BMI, BMI before delivery, BMI gain, and neonatal weight among different DRA phenotypes (*p* < 0.05), whereas no significant differences were found in height and gravidity (*p* > 0.05). Chi-square tests showed statistically significant differences in the mode of delivery, episiotomy, perineal laceration, and cesarean section during the trial of labor (*p* < 0.05), whereas no significant differences were found in assisted delivery, precipitous labor, and labor analgesia (*p* > 0.05). As the severity of DRA decreased (from 000 to 111), there were gradient changes in age (30–28 years), gestational days (278–272 days), weight/BMI gain (14.4–12 kg/5.8–4.7 kg), neonatal weight (3.2–3.0 kg), vaginal delivery (44.8–75.7%), episiotomy (11.5–20.4%), perineal laceration (32.3–46.7%), and cesarean section (55.2–24.3%). When age (29 years) and neonatal weight (3.1 kg) exceeded the median levels of the total population, there was a higher likelihood of complete separation of the recti abdominis muscles. When gestational age (274 days) and weight/BMI gain (13.4 kg/5.3) exceeded the median of the total population, there was a higher likelihood of separation at two or more sites of the recti abdominis muscles (Table 1).

### Multivariate logistic regression analysis of factors related to postpartum DRA phenotypes

After collinearity testing, the continuous variables included in the regression analysis were age, gravidity, gestational days, pre-pregnancy BMI, BMI gain, and neonatal weight. The categorical variables included the mode of delivery, episiotomy, perineal laceration, cesarean section during the first or second stage of labor, assisted delivery, precipitous labor, and labor analgesia. The study and correction factors are described in the Introduction section.

Although gradient changes were observed in the data from 000 to 111, the regression results showed that, compared with 111, the three most common subtypes of DRA had consistently associated variables (*p* < 0.05), which were not affected by factor correction. However, the variables associated with these subtypes differed from each other.

### Complete separation type (000)

In all five models, compared with type 111, type 000 was significantly associated with BMI gain (OR 1.21–1.33), neonatal weight (OR 2.25–3.99), and vaginal delivery (OR 0.13–0.16) (*p* < 0.05). When all correction factors were included (Model 5), the significance of neonatal weight and vaginal delivery disappeared. No significant association was found between type 000 and perineal injury or trial of labor (*p* > 0.05).

### Combined upper umbilical and periumbilical separation type (001)

Type 001 was significantly and consistently associated with neonatal weight (OR 1.92–2.85) and vaginal delivery (OR 0.18–0.22) (*p* < 0.05). When all correction factors were included (Model 5), it was also significantly associated with BMI gain (OR 1.11) (*p* < 0.05). When intrapartum correction factors were not included (Model 2/Model 3), type 001 was significantly associated with episiotomy (OR 2.59–3.13). When prenatal correction factors were not included (Model 2/Model 4), it was significantly associated with perineal lacerations (OR 2.22–2.53). No significant association was found between type 001 and trial of labor (*p* > 0.05).

### Isolated periumbilical separation type (101)

Type 101 was significantly and consistently associated with vaginal delivery (OR 0.18–0.21), episiotomy (OR 3.21–4.43), perineal laceration (OR 4.17–4.44), and cesarean section during the first stage of labor (OR 2.55–2.81) (*p* < 0.05). No significant association was found with BMI gain (*p* > 0.05). When prenatal correction factors were not included (Model 2/Model 4), type 101 was significantly associated with neonatal weight (OR 2.19–2.22).

**Figure 3.**
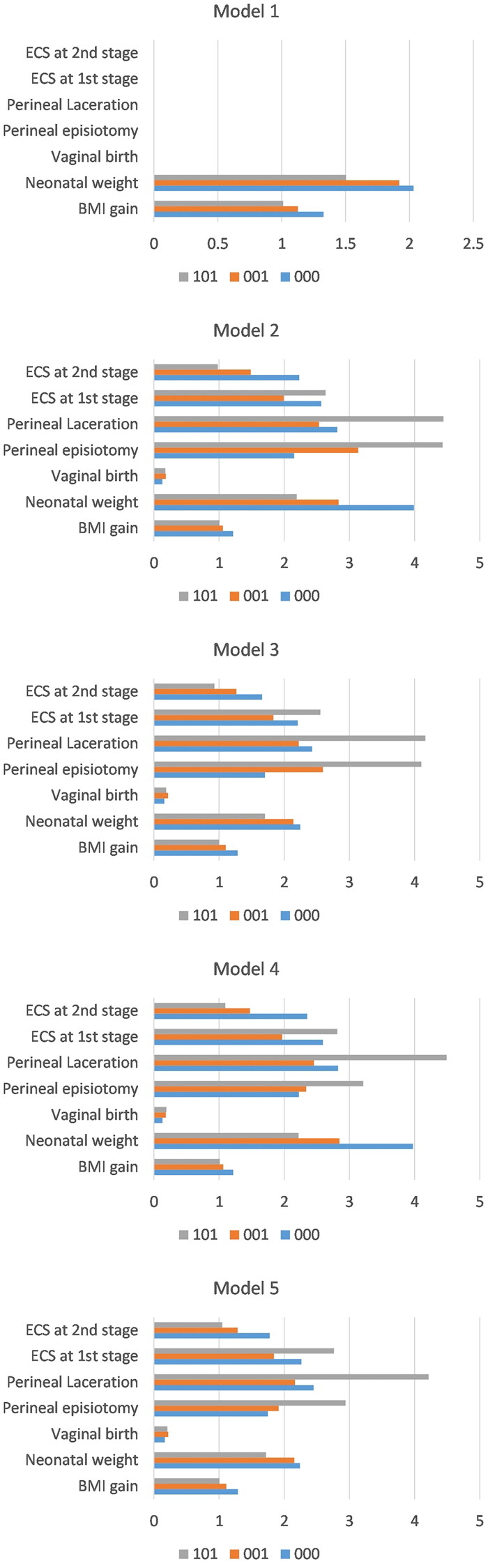
The odds ratio of factors influencing the rectic abdominis phenotype in different adjusted factor models. Model 1: Odds ratios (OR) of each prenatal factor after including prenatal correction factors. Model 2: OR of each prenatal and intrapartum factor without correction. Model 3: OR of each prenatal and intrapartum factor after including only prenatal correction factors. Model 4: OR of each prenatal and intrapartum factor after including only intrapartum correction factors. Model 5: OR of each prenatal and intrapartum factor after including all correction factors. Prenatal correction factors included age, gravidity, gestational days, and p-BMI. Intrapartum correction factors included assisted delivery, precipitous labor, and labor analgesia.

In summary, the regression results suggest that vaginal delivery is a protective factor against DRA. As the number of separation sites increases, the association between trial of labor and perineal injury and the phenotype weakens, whereas BMI and neonatal weight show the opposite trend. (see Table 3 and Figure 3 for detailed data.)

**Table 3.** Multivariate regression analysis of risk factors for postpartum Diastasis recti abdominis phenotypes (odds ratio results).

Type		BMI gain	Neonatal weight	Vaginal birth	Perineal episiotomy	Perineal laceration	ECS1st	ECS2nd
111		Reference	Reference	Reference	Reference	Reference	Reference	Reference
000	Model 1	*1.33	2.03	None	None	None	None	None
	Model 2	*1.21	*3.99	*0.13	2.15	2.81	2.57	2.23
	Model 3	*1.29	*2.25	*0.16	1.70	2.43	2.21	1.66
	Model 4	*1.22	*3.97	*0.13	2.23	2.83	2.59	2.35
	Model 5	*1.29	2.24	0.17	1.75	2.45	2.26	1.78
001	Model 1	*1.13	*1.92	None	None	None	None	None
	Model 2	1.06	*2.83	*0.18	*3.13	*2.53	1.99	1.49
	Model 3	1.10	*2.14	*0.22	*2.59	2.22	1.83	1.27
	Model 4	1.06	*2.85	*0.18	2.34	*2.46	1.97	1.47
	Model 5	*1.11	*2.15	*0.22	1.92	2.17	1.84	1.29
101	Model 1	1.01	1.50	None	None	None	None	None
	Model 2	1.00	*2.19	*0.18	*4.43	*4.44	*2.63	0.98
	Model 3	1.00	1.70	*0.19	*4.10	*4.17	*2.55	0.93
	Model 4	1.01	*2.22	*0.19	*3.21	*4.49	*2.81	1.10
	Model 5	1.01	1.72	*0.21	2.94	*4.22	*2.76	1.05

000: Complete separation type; 001: combined upper umbilical and periumbilical separation type; 010: combined upper umbilical and lower umbilical separation type; 011: isolated upper umbilical separation type, 100: combined periumbilical and lower umbilical separation type, 101: isolated periumbilical separation type, 110: isolated lower umbilical separation type, 111: no separation type, ECS1st: cesarean section during the first stage of labor, ECS2nd: cesarean section during the second stage of labor. *Notes*. 1.* indicates *p* < 0.05. 2. Model 1: Odds ratios (OR) of each prenatal factor after including prenatal correction factors. Model 2: OR of each prenatal and intrapartum factor without correction. Model 3: OR of each prenatal and intrapartum factor after including only prenatal correction factors. Model 4: OR of each prenatal and intrapartum factor after including only intrapartum correction factors. Model 5: OR of each prenatal and intrapartum factor after including all correction factors. 3. The prenatal correction factors included age, gravidity, gestational days, and p-BMI. The intrapartum correction factors included assisted delivery, precipitous labor, and labor analgesia.

## Discussion

Our retrospective study found that almost 90% of singleton primiparous women had DRA 42 days postpartum. The most common presentation was periumbilical separation, followed by upper umbilical separation, and lower umbilical separation was the least common presentation. Upper umbilical separation typically coexists with periumbilical separation, and lower umbilical separation almost always coexists with both upper and periumbilical separations. Vaginal delivery was identified as a protective factor against DRA. Both prenatal and intrapartum factors were associated with DRA phenotypes; however, the specific factors varied across the different subtypes.

### Postpartum diastasis recti abdominis phenotypes

Corvino et al. [13] conducted a DRA phenotype study on 92 women aged 30–61 years and found that 89.1% of the subjects had DRA, with five phenotypes identified and isolated upper umbilical separation being the most common (58.5%), followed by upper gap-dominant complete separation (29.3%). In our study, to better reflect the impact of the first childbirth on the recti abdominis muscles, we selected singleton primiparas as the study population. In postpartum abdominal ultrasound reports over one year, the proportion of DRA in singleton primiparous women was remarkably close to that reported by Corvino (89.4% vs. 89.1%, respectively). Among the eight potential phenotypes in binary combinations, types 001 and 101 were the main DRA phenotypes at 42 days postpartum in primiparous women, accounting for 81.5%, with type 001 being the most prevalent (47.8%). Types 000 and 111 accounted for 6.7% and 10.6%, respectively, showing that the periumbilical region was the most susceptible to separation after the first childbirth, followed by the upper umbilical region. The proportion of complete separation was not as high as reported by Corvino, suggesting that separation of the lower umbilical part of the recti abdominis may be related to age and parity. Upper umbilical separation almost always coexists with periumbilical separation, and lower umbilical separation coexists with upper and periumbilical separations, with the separation ratio decreasing in this order. This shows that separation is most likely to occur in the periumbilical region, followed by the upper umbilical region, and finally progressing to complete separation. Additionally, the three separation phenotypes, namely the combined upper and lower umbilical separation type (010), combined periumbilical and lower umbilical separation type (100), and isolated lower umbilical separation type (110), were extremely rare and could not be analyzed statistically. In the case data analysis, we did not find extreme changes in prenatal and intrapartum indicators; therefore, anatomical variations cannot be excluded.

### Factors related to postpartum diastasis recti abdominis

Previous studies have reported that multiparity, cesarean section, increased BMI, diabetes, and waist-to-hip ratio are associated with postpartum DRA [9,24–28]; however, no study has reported on intrapartum DRA. We believe that evidence from the prenatal and intrapartum periods can help in the development of effective prevention strategies for DRA. Our data confirmed that the mode of delivery and BMI gain are factors that affect the occurrence of DRA. Currently, there is a social notion that cesarean sections help reduce the incidence of postpartum DRA. This perception, which may be based on the idea that vaginal delivery can affect the abdominal muscles and worsen separation, while cesarean section can involve suturing of the recti abdominis muscle, has led some pregnant women to request an elective cesarean section. Neither earlier studies [13,20] nor our results support this notion, as no type of DRA was found to be reduced by cesarean sections. In our study unit, for cesarean sections performed with a transverse abdominal incision, rectus abdominis suturing is performed; however, we did not find an increased proportion of cesarean sections in types 001, 011 (data not shown), and 111. In contrast, the proportion of cesarean sections was highest for type 000 clusters. In all our regression models, vaginal delivery was negatively correlated with DRA.

Our study assumed that the occurrence of DRA is due to changes in IAP experienced by the recti abdominis, including IAP increases due to uterine enlargement during pregnancy and active abdominal muscle contractions during labor. Therefore, maternal/fetal weight increase, mode of delivery, perineal injury, and labor trial experience were the main assessment indicators in this study. The results showed that these indicators were associated with the DRA. At the same time, we did not find that pregnancy history before 20 weeks was related to postpartum DRA.

We found that the delivery impact factors for the different types of DRA were not identical. Compared to type 111, types 000 and 001 are associated with BMI gain and neonatal weight, which is attributed to the increase in IAP caused by the increase in maternal/fetal weight. In addition to the common influencing factors mentioned above for type 000, type 001 was also significantly related to episiotomy when intrapartum correction factors (Model 2/Model 3) were not included, and significantly related to perineal laceration when prenatal correction factors (Model 2/Model 4) were not included. This suggests that separation below the umbilicus is related to episiotomy or laceration; however, these effects appear to have some interactive relationships with intrapartum or prenatal correction factors. This conjecture was verified in type 101, which was related to neonatal weight, episiotomy or laceration, and cesarean section during the first stage of labor in all models. In clinical practice, episiotomy is performed to reduce perineal resistance, and laceration may be related to improper perineal protection; however, it is more often because the perineal elasticity cannot adapt to the degree of expansion required for the expulsion of the fetal head. Therefore, both episiotomy and laceration can be considered changes made to reduce resistance, which seems to be beneficial for protecting the lower part of the rectus abdominis muscle from separation (001/101).

However, this does not explain why vaginal delivery was negatively correlated with the incidence of DRA in this study. If reducing outlet resistance can protect against abdominal muscle separation, a cesarean section should be considered a protective factor. During labor, abdominal muscle contractions form a counterforce to the enlarged uterus, which appears to further increase abdominal pressure and worsen muscle separation. However, our results do not support this hypothesis. The two recti abdominis muscles are connected by the linea alba, and Brauman [29] found that the stretch range of the linea alba is limited. The degree of DRA does not have a constant relationship with postpartum abdominal protrusion, and it is believed that DRA is not only due to the stretching of the linea alba but also due to the stretching of the entire abdominal wall. Fan et al. [20] found that in women who had cesarean section, the recti abdominis spacing increased, with partial abdominal fascia thickening and recti abdominis thinning, while in women who had vaginal delivery, localised rectus abdominis thinning mainly occurred. These two studies suggest that the abdominal muscles are stretched and thinned after childbirth, but the changes in the fascia depend on the mode of delivery. Vaginal delivery appears to prevent these changes. In addition, Cintesun et al. [30] found that suturing the rectus abdominis muscle during a cesarean section did not affect the thickness and spacing of the abdominal muscles in the surgical area. This explains why women who underwent cesarean sections did not benefit from postpartum abdominal muscle training, even if their abdominal muscles were sutured during the surgery. Gunnarsson et al. [31] found in preoperative assessments of patients with DRA that there was a significant negative correlation between the degree of separation of the lower part of the recti abdominis and the strength of abdominal isometric contractions; however, no such relationship was found in the upper part of the recti abdominis. They believe that the lower part of the rectus abdominis plays a key role in maintaining core stability. Combining the above data from previous studies, we further attempt to explain that although the labor process is relatively short compared to the period from pregnancy to childbirth, the only period when the abdominal muscles undergo the greatest isometric contractions is the second stage of labor, during which active contractions (isometric contractions) help strengthen the connections between the abdominal muscles (lower umbilical region). In addition, regular uterine contractions during the first stage of labor may also have some effects, leading to similar strengthening effects on the abdominal muscle connections (upper umbilical region).

### Significance of multiple model analysis

In this study, we used five models to evaluate the factors related to DRA, which not only excluded the influence of confounding factors but also aided in clinical application. In actual working scenarios, we may face the following challenges: predicting DRA prenatally and providing interventions, preventing DRA during labor, and providing DRA screening recommendations for postpartum women in resource-limited settings. Our five models were set up with reference to these scenarios. During the prenatal stage, attention should be paid to monitoring and controlling maternal and fetal weight. During labor, rational selection of episiotomy is helpful in protecting the upper part of the abdominal muscles. For women with an increase in BMI greater than 5 during pregnancy and neonatal estimated weight exceeding 3 kg, rational selection of episiotomy and timing of elective cesarean section should be considered, and abdominal muscle screening should be prioritized postpartum.

In summary, regarding the prevention of postpartum DRA, our study results indicate that all pregnant women, especially those lacking the prerequisites for vaginal delivery (such as breech presentation, placenta previa, or other contraindications), should manage their weight throughout pregnancy. For women who satisfy the requirements for vaginal delivery, a trial of labor should be encouraged, as even if it fails, it still helps reduce the severity of the DRA. Additionally, the rational use of episiotomies during childbirth is advocated.

### Limitations of the study and future directions

The retrospective nature of this study limits the determination of the incidence of various DRA phenotypes. Therefore, well-designed prospective studies are urgently warranted to determine the prevalence of postpartum DRA phenotypes and clarify the causal relationships between these phenotypes and their related risk factors. In addition, although this study verified and identified prenatal and intrapartum factors related to postpartum DRA phenotypes, the underlying pathophysiological mechanisms require further investigation in future studies. Nevertheless, this study, based on evidence from earlier research, may help guide the development of prenatal, intrapartum, and postpartum interventions.

Currently, the main treatment for DRA involves postpartum rehabilitation exercises. There is evidence that targeted abdominal muscle training can improve DRA symptoms [32–37]. Different exercise postures may have different effects on different parts of separation [38]. Our study found that labor trials help reduce the occurrence of DRA, suggesting that prenatal abdominal interventions may serve as a strategy to prevent postpartum DRA.

## Conclusion

DRA is common in abdominal ultrasound examinations at 42 days postpartum, with significant separation seen in the periumbilical and upper umbilical regions. The risk factors for the different DRA subtypes vary widely between studies. Careful weight management during pregnancy, advocacy for spontaneous vaginal delivery, and rational use of episiotomies may be strategic interventions to reduce the incidence of DRA.

## Data Availability

The datasets used and analyzed in this study are available from the corresponding author upon reasonable request.
